# Phosphoinositide 3-Kinase regulates glycolysis through mobilization of Aldolase A from the actin cytoskeleton

**DOI:** 10.1186/2049-3002-2-S1-P86

**Published:** 2014-05-28

**Authors:** Hai Hu, Ashish Juvekar, Costas Lyssiotis, John G Albeck, Dean R Tolan, John M Asara, Gerburg M Wulf, Lewis C Cantley

**Affiliations:** 1 Medicine, Beth Israel Deaconess Medical Center, Boston, MA, USA; 2 Medicine, Weil Cornell Medical College, New York, NY, USA; 3 Biology, Boston University, Boston, NY, USA; 4 University of California, Davis, CA, USA

## Background

Phosphoinositide 3-Kinase (PI3K) has been shown to modulate multiple steps in glucose uptake and metabolism through activation of the protein kinase, AKT. In order to dissect the contributions of PI3K-pathway components, we examined the effects of specific enzyme inhibitors on the regulation of glycolysis.

## Methods

We measured reduction of NAD to NADH, occurring at the GAPDH step, as a read-out for glycolysis in living cells; mass spectroscopy to determine the relative abundance of glycolytic metabolites in breast cell cultures and in a mouse model of breast cancer; immunoblotting and confocal live cell microscopy to delineate the intracellular signaling cascade downstream from PI3K and a spectrophotometric assay to determine Aldolase activity.

## Results

In breast epithelial cells PI3K-, but not AKT-, SGK- or mTOR-inhibitors cause a significant decrease in glycolysis at the step catalyzed by Aldolase A. We show that growth factors stimulate Aldolase A release from the actin cytoskeleton and an increase in cellular Aldolase activity in a PI3K dependent manner. The mobilization and activation of Aldolase is dependent on Rac1-catalyzed phosphorylation of p-21 activated kinase (PAK) and subsequent mobilization of the actin cytoskeleton.

## Conclusions

This newly identified AKT- and mTOR-independent role of PI3K in controlling glucose metabolism has important implications in regard to utilization of PI3K pathway inhibitors for treatment of epithelial cancers.

